# COVID-19 Vaccination Did Not Change the Personal Protective Behaviors of Healthcare Workers in China

**DOI:** 10.3389/fpubh.2021.777426

**Published:** 2021-12-21

**Authors:** Nan Zhang, Hao Lei, Li Li, Tianyi Jin, Xiyue Liu, Doudou Miao, Boni Su, Zhongming Bu, Lin Fan, Peng Xue, Jingchao Xie, Yuguo Li

**Affiliations:** ^1^Beijing Key Laboratory of Green Built Environment and Energy Efficient Technology, Beijing University of Technology, Beijing, China; ^2^School of Public Health, Zhejiang University, Hangzhou, China; ^3^China CDC Key Laboratory of Environment and Population Health, National Institute of Environmental Health, Chinese Center for Disease Control and Prevention, Beijing, China; ^4^Department of Clean Energy Research, China Electric Power Planning and Engineering Institute, Beijing, China; ^5^Department of Energy and Environmental System Engineering, Zhejiang University of Science and Technology, Hangzhou, China; ^6^Department of Mechanical Engineering, The University of Hong Kong, Pokfulam, Hong Kong SAR, China; ^7^School of Public Health, Li Ka Shing Faculty of Medicine, The University of Hong Kong, Pokfulam, Hong Kong SAR, China

**Keywords:** COVID-19, healthcare worker (HCW), personal protective behavior, vaccination, transmission route, mask, hand hygiene, indoor ventilation

## Abstract

Personal protective behaviors of healthcare workers (HCWs) and dynamic changes in them are known to play a major role in the hospital transmission of severe acute respiratory syndrome coronavirus 2 (SARS-CoV-2). In this study, 1,499 HCWs in Chinese hospitals completed an online survey about their knowledge on SARS-CoV-2 transmission and their personal protective behaviors before and after coronavirus disease 2019 (COVID-19) vaccination. Of all the respondents, 89% were vaccinated at the time of the survey and 96% believed that the vaccine was effective or highly effective. Further, 88% of the vaccinated HCWs expressed that they would get revaccinated if the vaccination failed. Compared with HCWs with a lower education level, those with a higher education level had less fear of being infected with SARS-CoV-2 and reported a lower negative impact of the pandemic on how they treated patients. Physicians and nurses were willing to believe that short-range airborne and long-range fomite are possible transmission routes. HCWs with a higher education level had a better knowledge of COVID-19 but worse personal protective behaviors. The fact that HCWs with a longer work experience had worse personal protective behaviors showed that HCWs gradually relax their personal protective behaviors over time. Moreover, vaccination reduced the negative effects of the COVID-19 pandemic on how the HCWs treated patients. Importantly, the survey revealed that after vaccination, HCWs in China did not relax their personal protective behaviors, and it may bring a low potential risk for following waves of variant virus (e.g., delta).

## Introduction

Sustained and adequate personal protective behaviors have been instrumental in tackling the coronavirus disease 2019 (COVID-19) pandemic (1, 2). As of the end of November, 2021, more than 250 million people have been infected with severe acute respiratory syndrome coronavirus 2 (SARS-CoV-2), the causal pathogen, and over 5.1 million have died from the infection (https://covid19.who.int/).

Most cases of transmission have occurred between people in indoor settings, and personal protective behaviors has played a major role in transmission (3). Following a record-breaking speed in vaccine development, multiple COVID-19 vaccines have become available (4). As of August 12, 2021, more than 4.4 billion vaccine doses had been administered worldwide (https://covid19.who.int/). However, it remains unclear whether people change their personal protective behaviors against COVID-19 after being vaccinated.

Compared with the general public, healthcare workers (HCWs) are more vulnerable to COVID-19 infection during the ongoing pandemic because of frequent contacts with confirmed patients and a higher risk of meeting potentially infected patients (5). In China, 4.4% of all COVID-19 cases were in HCWs (6). In the USA, 9,282 HCWs were infected with SARS-CoV-2 from February 12 to April 9, 2020, which constituted 19% of the total infected cases in the country at that time (7). In the early phase of the pandemic, up to 10% of HCWs in some countries were infected with SARS-CoV-2 (8). Compared with the general public, HCWs have maintained a higher level of perceived risk and worry about the COVID-19 crisis and better adherence to personal preventive practices (9).

Because of their high susceptibility to infection, it is strongly recommended that HCWs should take sufficient protective equipment to minimize the infection risk (10). Mask and gloves wearing were primary suggested for risk reduction of HCWs and the use of a mask by the infected was very effective (11). Except for sufficient personal protective equipment, HCWs are prioritized for vaccination (12). Studies have evaluated the efficacy of vaccines (13) and the role of vaccination in the prevention and control of both COVID-19 and influenza in hospital settings (14). However, no study has investigated the changes in the personal protective behaviors of HCWs before and after vaccination. For example, it remains unclear whether, after vaccination, HCWs continue taking all precautions while treating patients or relax their preventive measures. The latter case may greatly increase their susceptibility to the newer variants of the virus (e.g., alpha and delta) given that the efficacy of current vaccines against newer variants is potentially low (15). Therefore, understanding post-vaccination changes in the protective behaviors of HCWs is crucial.

In this study, we performed an online survey of HCWs in China to understand the levels of their COVID-19 transmission knowledge and personal protective behaviors. We also analyzed changes in their personal protective behaviors after vaccination. The results are expected to inform COVID-19 prevention and control efforts at hospitals.

## Methods

### Participants and Procedures

An online survey was conducted in HCWs from Chinese hospitals on June 19 and 20, 2021. All of the respondents were at work in a hospital at the time of the survey and completed the survey within that day. Vaccinated respondents were asked to answer 34 questions, whereas non-vaccinated respondents were asked to answer 27 questions. A questionnaire was considered invalid if the respondent submitted all responses within 2 min, the answer to the test question “Please select the third option” was wrong, or the respondent was not an HCW. Of the 1,742 collected questionnaires, 243 were invalid. Thus, the remaining 1,499 valid questionnaires were analyzed.

The survey was conducted using a professional software application named Wenjuanxing (www.wjx.cn) and distributed to HCWs through several communication groups on WeChat, the most widely used messaging and social media app in China. Some HCWs were directly recruited in these communication groups, who further disseminated the online questionnaire to their colleagues to complete the survey. The survey took approximately 5 min to complete, and the respondents received CNY 10 after successfully submitting a valid questionnaire. This survey was approved by the Ethics Committee of Beijing University of Technology (HS202111001).

### Scope of the Survey Questions

The survey included questions about demographic information, non-individual COVID-19 prevention and control measures adopted in their hospitals, knowledge about COVID-19, and changes in personal protective behaviors after vaccination. The following demographic data were collected: gender, age, education level, annual income, occupation in the hospital (physician, nurse, administrative staff, cleaner, and others), province of the workplace, department in the hospital [high-risk departments include respiratory, ophthalmology and otorhinolaryngology, anesthesiology, infectious disease, general, and ICU departments (16, 17)], hospital classification (as per the 3-tier system—with 3 being the highest level—adopted by the Ministry of Health; each level is subdivided into grades A, B, and C in the following order [highest to lowest]: 3A+, 3A, 3B, 3C, 2A, 2B, 2C, 1A, 1B, 1C, and <1C), years of work experience, and vaccination status. No question soliciting information of personal identification was included in the questionnaire. The non-individual COVID-19 prevention measures (in the hospitals) solicited by the questions included convenience of hand washing, indoor ventilation in the workplace, and guidelines for COVID-19 prevention and control in the hospital and department. Knowledge about COVID-19 solicited by the questions included vaccination efficacy, possible SARS-CoV-2 transmission routes, intention to be revaccinated in case of vaccination failure, and changes in protective behaviors due to outbreaks in other cities. Personal protective behaviors before and after vaccination solicited by the questions included the hand washing frequency, desk surface cleaning, air disinfection, wearing protective gear in the hospital, paying attention to indoor ventilation, fear of contracting COVID-19, and negative impact of the pandemic on how they treated patients.

At the time point (June 30, 2021) of questionnaire distribution, there were 91,846 cumulative reported COVID-19 cases and 124 confirmed new infected cases (both local and imported) within the past week (June 24 to 30, 2021) (Supplementary Figure 1). The COVID-19 pandemic almost kept the stable (26.1 ± 29.9 daily new reported cases) after the vaccination since January 1, 2021, and majority of the infected were imported cases. Therefore, we considered that there is no serious influence on personal protective behavior of HCWs by the infection at that time.

### Data Analysis

The descriptive statistics of the HCW's protective behaviors are presented as means, medians, and frequencies. When processing the data, we first validated the normality of distribution. If the data were normally distributed, the independent samples *t*-test was used to calculate correlations between two factors. Otherwise, the Kruskal–Wallis test was used. Wilcoxon's two-sample *t*-test was used for correlation analyses of changes in protective behaviors due to vaccination. All of the statistical analyses were performed using SPSS 26.0 (IBM). A two-tailed *p*-value < 0.05 was considered to indicate statistical significance. Only the factors significantly correlated with the dependent variables were analyzed in this study.

## Results

**Figure 1:**
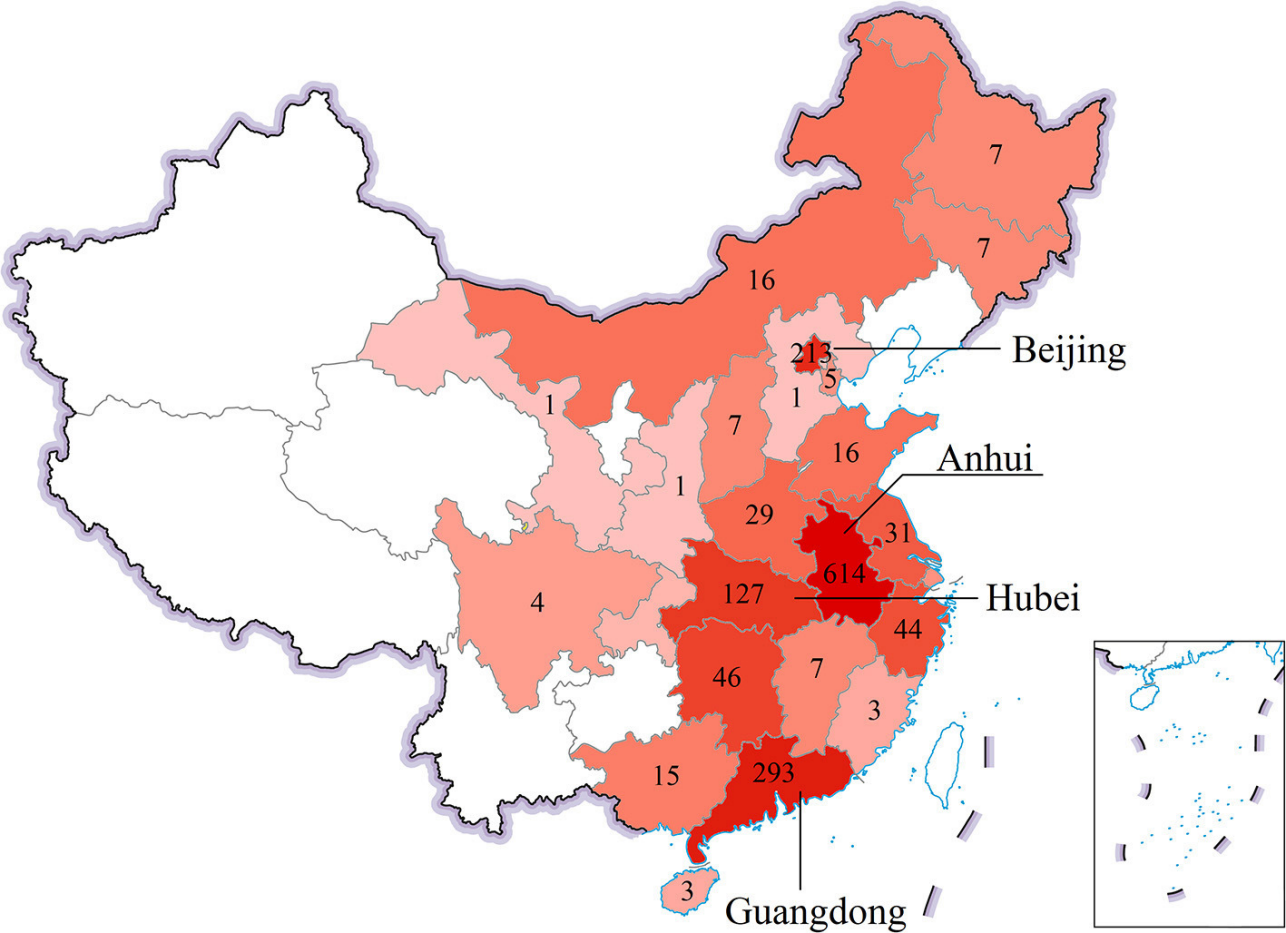
Spatial distribution of the respondents.

Of the 1,499 respondents who submitted valid questionnaires, 1,124 (75%) were female. More than 43% (646/1,499) of the respondents were 26 to 35 years old. More than 80% (1,247/1,499) of respondents were from Beijing, Guangdong, Hubei, and Anhui provinces (Figure 1). Nearly 90% (1,334/1,499) of the respondents had a Bachelor's/college or higher degree from a university or junior college. One-third of the respondents earned < 50,000 CNY (7,825 USD) per year, and one-third earned more than 100,000 CNY (15,650 USD) per year. Of all 1,499 respondents, 42, 30, 9, 2, 2, and 15% were nurses, physicians, administrative, cleaning personnel, technician, and other occupations, respectively. Because of large number of administrative and possible vulnerable occupation of cleaning personnel, we only considered physicians, nurses, administrative, and cleaning personnel when analyzing the behavior by occupation. For physicians and nurses, 22% (334/1,499) worked in high-risk departments during the pandemic. Around half of the respondents (726/1,497) had a work experience of <10 years, and 89% of 1,499 respondents were vaccinated (at least received one dose). The demographic information of the 1,499 respondents is provided in Supplementary Table 1, Figure 1.

### Vaccination Rates

Of all the respondents, 88.9% (1,332/1,499) were vaccinated at the time of the survey (Table 1). Vaccination was significantly associated with gender, age, annual income, and province. Male HCWs had a higher vaccination rate (92.8%, 348/375) than female HCWs (87.5%, 984/1,124). HCWs aged 26–35 years had the lowest vaccination rate (84.4%, 545/646). HCWs in high-urbanization areas, i.e., Beijing [urbanization rate (UR) = 86.5%] and Guangdong (UR = 70.7%), had a higher mean vaccination rate (92.5%, 467/505) than those in low-urbanization areas (mean vaccination rate of 87.2%, 647/742), i.e., Hubei (UR = 60.3%) and Anhui (UR = 54.7%). No significant association was observed between the vaccination rate and other factors including education, occupation, annual income, years of work experience, hospital classification, and hospital departments.

**Table 1:** Distribution of vaccination rates among healthcare workers by demographic characteristics.

**Personal attribute**	**Vaccinated**	**Not vaccinated**	***p*** **-value**
Total, *N* = 1,499 (%)	1,332 (88.9%)	167 (11.1%)	-
Gender, *n* = 1,499 (%)	-	-	0.005
Male	348 (92.8%)	27 (7.2%)	**-**
Female	984 (87.5%)	140 (12.5%)	**-**
Age, *n* = 1,499 (%)	-	-	<0.001
≤ 25 years	207 (92.0%)	18 (8.0%)	**-**
26–35 years	545 (84.4%)	101 (15.6%)	**-**
36–45 years	355 (92.4%)	29 (7.6%)	**-**
>45 years	225 (92.2%)	19 (7.8%)	**-**
Province, *n* = 1,247 (%)			0.016
Beijing (*n* = 213)	194 (91.7%)	19 (8.3%)	
Guangdong (*n* = 292)	273 (93.5%)	19 (6.5%)	
Hubei (*n* = 127)	108 (85.0%)	19 (15.0%)	
Anhui (*n* = 615)	539 (87.6%)	76 (12.4%)	

### Personal Protective Behaviors

HCWs washed their hands, cleaned their desk surface, and disinfected the indoor air an average of 12.24, 2.81, and 2.44 times per day, respectively (Table 2). More than half (53.0%, 706/1,332) of the HCWs paid an extreme high level of attention to indoor ventilation, whereas, none of them paid very little attention. HCWs who reported a higher frequency of hand washing were significantly more likely to report higher frequencies of surface disinfection (*p* <0.001) and air disinfection (*p* < 0.001). Male HCWs washed their hands an average of 10.33 times per day during the pandemic, a value 9.1% lower than the average frequency among female HCWs (11.36 times per day). Younger HCWs aged <35 years had a higher frequency of air disinfection (2.60 times per day), but a lower frequency of hand washing (11.82 times per day) and paid less attention to indoor ventilation, in comparison with HCWs aged over 35 years (hand washing: 12.78 times per day; air disinfection: 2.24 times per day). Nurses washed their hands an average of 15.33 times per day, which was 34.9% higher than the average frequency among physicians (11.36 times per day). HCWs who had a longer work experience washed their hands more frequently and paid more attention to indoor ventilation, but less frequently disinfected the indoor air. HCWs who worked in lower-level hospitals (Grade 1 and lower) had the worst personal protective behavior (Supplementary Table 2). Frequencies of surface and air disinfection were 31 and 36% higher, respectively, among HCWs who worked in high-risk departments than among those who worked in other departments.

**Table 2:** Distribution of personal protective behaviors among healthcare workers by demographic characteristics.

**Personal attribute**	**Hand washing**	**Surface disinfection**	**Air disinfection**	**Indoor ventilation^a^**
	**(times/day)**	**(times/day)**	**(times/day)**	
Total	12.24	2.81	2.44	3.43
Gender^b^	*p* < 0.001	*p* = 0.074	*p* = 0.148	*p* = 0.369
Male	10.33	-	-	-
Female	11.36	-	-	-
Age (years)	*p* = 0.25	*p* = 0.233	*p* < 0.001	*p* = 0.009
≤ 25	11.74	-	3.16	3.39
26–35	11.85	-	2.38	3.37
36–45	13.08	-	2.39	3.45
>45	12.31	-	2.00	3.55
Education	*p* < 0.001	*p* < 0.001	*p* < 0.001	*p* = 0.010
High school or lower	9.58	4.07	4.01	3.58
University/junior college	12.87	2.76	2.36	3.43
Master's or higher	11.23	2.19	1.73	3.31
Annual income (USD)	*p* < 0.001	*p* = 0.001	*p* < 0.001	*p* = 0.827
≤ 7,825	11.08	3.30	2.90	-
7,826–15,650	12.91	2.64	2.38	-
15,651–31,300	12.87	2.73	2.11	-
>31,300	11.74	2.22	2.13	-
Occupation	*p* < 0.001	*p* = 0.072	*p* < 0.001	*p* = 0.254
Physician	11.36	-	1.95	-
Nurse	15.33	-	2.39	-
Administrative staff	9.32	-	3.11	-
Cleaner	8.52	-	3.29	-
Years of work experience	*p* < 0.001	*p* = 0.468	*p* = 0.009	*p* = 0.013
<5	10.93	-	2.62	3.37
5–10	11.94	-	2.55	3.39
10–20	13.44	-	2.47	3.46
>20	13.05	-	1.95	3.53
Department, *n* = 1,332	*p* = 0.148	*p* < 0.001	*p* < 0.001	*p* = 0.001
High-risk (400)	-	3.4	3.0	1.5
Others (932)	-	2.6	2.2	1.6

a *On a scale of 1 to 5 where 1 indicates very little attention and 5 indicates a high level of attention to indoor ventilation.*

b *Because more than 97% of nurses and more than 70% of cleaners and technicians were female, we considered physicians and administrative staff only when analyzing the correlation between gender and personal protective behaviors*.

During the pandemic, 92.7, 50.8, 17.3, 15.0, 11.4, and 8.1% of 1,499 HCWs wore surgical masks, gloves, N95 respirator, protective clothing, face shield, and goggles, respectively, during most of their time in hospitals (Supplementary Table 3). Personal protective behaviors in the hospitals were not associated with gender. Younger HCWs (≤ 25 years) adopted a higher level of protective measures than older HCWs (>25 years). More physicians (18.2%, 83/455) wore N95 respirators than nurses (12.0%, 75/624) during the pandemic. HCWs with a longer work experience adopted a lower level of protective measures. Only 87.9% (236/268) and 45.2% (121/268) of the HCWs who worked in lower-level hospitals (Grade 1 and lower) wore surgical masks and gloves most of time during the pandemic (Supplementary Table 3). HCWs who worked in high-risk departments had a 2-time higher use rate of N95 respirators, face shields, protective clothing, and goggles than those worked in other departments.

Only 9.3% (139/1,499) of the HCWs reported that they had no fear of being infected in the hospital, and only 5.1% (76/1,499) reported that they treated the patients as usual during the pandemic (Table 3). Younger HCWs were more easily influenced by the pandemic. Compared with HCWs with a lower education level, those with a higher education level had less fear of being infected and reported a lower negative impact of the pandemic on how they treated patients. Cleaners reported the most fear of being infected, whereas administrative staff showed the least fear of being infected because they never needed to meet the patients directly. Physicians were less afraid of being infected than nurses. Other factors including gender, years of work experience, hospital classification, and departments showed no significant correlation with the HCW's fear of being infected or with the negative impact of the pandemic on how they treated patients.

**Table 3:** Distribution of healthcare worker's fear of being infected with SARS-CoV-2 and negative impact of the COVID-19 pandemic on how they treated patients, by demographic characteristics.

**Personal attribute**	**Fear of being**	**Negative impact on treatment**
	**infected^a^**	**of patients^b^**
Total	2.74	2.65
Age (years)	*p* = 0.309	*p* = 0.028
≤ 25	-	2.47
26–35	-	2.69
36–45	-	2.74
>45	-	2.62
Education	*p* = 0.020	*p* = 0.001
High school or lower	2.67	2.43
University/junior college	2.70	2.65
Master's or higher	2.89	2.85
Annual income (USD)	*p* < 0.001	*p* = 0.466
≤ 7,825	2.60	-
7,826–15,650	2.64	-
15,651–31,300	2.86	-
>31,300	3.09	-
Occupation	*p* < 0.001	*p* = 0.041
Physician	2.88	2.71
Nurse	2.61	2.70
Administrative staff	3.01	2.48
Cleaner	2.09	2.15

a *On a scale of 1 to 5 where 1 means that the respondent is extremely afraid of being infected in the hospital and 5 means that the respondent has no worry during the pandemic.*

b *On a scale of 1 to 5 where 5 means that the pandemic has no negative impact on the respondent's treatment of patients and 1 means that the pandemic has an extremely negative impact on the respondent's treatment of patients*.

**Figure 2:**
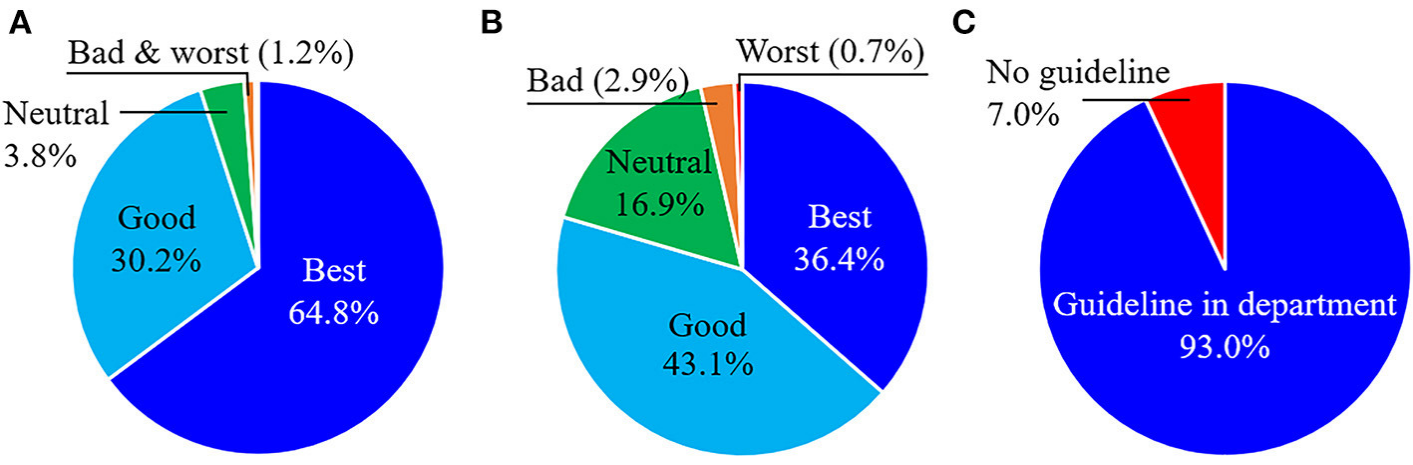
Analysis of COVID-19 prevention and control guidelines at Chinese hospitals. Healthcare worker's satisfaction with the **(A)** convenience of facilities for hand washing, **(B)** ventilation of indoor spaces, and **(C)** specific guidelines on COVID-19 prevention and control in the department.

In hospitals where the convenience of facilities for hand washing was highest, HCWs washed their hands an average of 13.0 times per day, which is 20% more than the average frequency in other hospitals (10.8 times per day). Compared with the facilities for hand washing, HCWs were more dissatisfied with the indoor ventilation in their hospitals (Figures 2A,B). Nearly 97% (433/447) of the HCWs working in high-risk departments reported that their departments had specific guidelines on COVID-19 prevention and control measures; this percentage was significantly higher than that in other departments (91%, 961/1,052) (Figure 2C).

### Personal Knowledge of COVID-19

**Figure 3:**
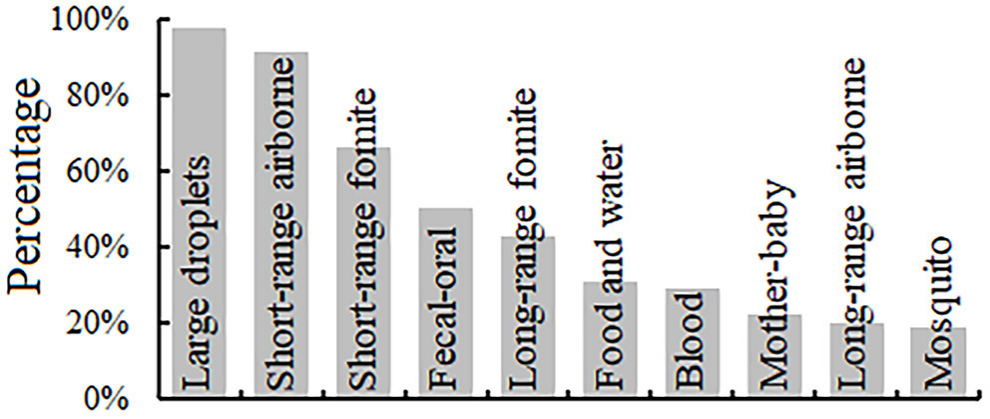
Percentages of healthcare workers who consider different routes to be likely for SARS-CoV-2 transmission.

Of 1,499 respondents, HCWs believed that the large-droplet (98.0%), short-range airborne (91.7%), short-range fomite (e.g., handshaking) (66.5%), and fecal–oral routes (50.5%) were the four main transmission routes of SARS-CoV-2 (Figure 3). Only 20.0% of the HCWs believed that long-range airborne was a dominant transmission route of SARS-CoV-2. The data provided in Supplementary Table 4 show that more female HCWs than male HCWs believed that short-range fomite was a possible transmission route. Compared with HCWs with a lower education level, those with a higher education level were more willing to believe that large-droplet, short-range airborne, and long-range fomite are possible transmission routes. Compared with other administrative staff and cleaners, physicians and nurses were more willing to believe that short-range airborne and long-range fomite were possible transmission routes. No significant correlation was found between the departmental risk classification and the HCW's understanding of the possible transmission routes of COVID-19.

As shown in Table 4, 95.9% (1,438/1,499) of the HCWs believed that the vaccine was effective or highly effective, and 88.1% (1,173/1,332) of the vaccinated HCWs expressed that they would get revaccinated if the current vaccination failed. Additionally, 60.2% (902/1,499) of the HCWs indicated that outbreaks in other cities would influence their personal protective behaviors. HCWs with a higher education level and a higher annual income more readily questioned the efficacy of the vaccine and were more easily influenced by outbreaks in other cities compared with their counterparts. All of the cleaners (100%) expressed that they would get revaccinated in case of vaccination failure. The HCWs who worked in higher-level hospitals and high-risk departments more readily questioned the efficacy of vaccine than their counterparts. No significant correlation was found between personal beliefs about COVID-19 and other factors including gender, age, and years of work experience.

**Table 4:** Distribution of healthcare worker's personal beliefs about COVID-19 by personal and hospital attributes.

**Personal and**	**Efficacy of**	**Injection again**	**Influenced by**
**hospital attributes**	**after vaccine^a^**	**after vaccine**	**outbreak in**
		**failure**	**cities**
Total	1.37	88.1%	60.2%
Education	*p* < 0.001	*p* = 0.003	*p* = 0.005
High school or lower	1.27	94.0%	49.7%
University/junior college	1.36	88.3%	60.7%
Master's or higher	1.50	82.9%	65.4%
Annual income (USD)	*p* = 0.001	*p* = 0.003	*p* = 0.400
≤ 7,825	1.31	90.6%	-
7,826–15,650	1.37	90.0%	-
15,651–31,300	1.40	85.5%	-
>31,300	1.51	80.4%	-
Occupation	*p* = 0.162	*p* = 0.028	*p* = 0.692
Physician	-	84.7%	-
Nurse	-	87.1%	-
Administrative staff	-	93.4%	-
Cleaner	-	100.0%	-
Hospital classification	*p* = 0.002	*p* = 0.063	*p* = 0.272
Grade 3A and higher	1.44	-	-
Grade 3B and 3C	1.38	-	-
Grade 2	1.38	-	-
Grade 1 and lower	1.27	-	-
Hospital department	*p* = 0.016	*p* = 0.305	*p* = 0.906
High-risk	1.40	-	-
Others	1.31	-	-

a *On a scale of 1 to 5 where 1 means the respondents believed that the efficacy of the vaccine was very high, while 5 means the respondents believed that the efficacy was negligible*.

### Changes in Personal Protective Behaviors Against COVID-19 After Vaccination

We found that the HCWs did not relax their personal protective behaviors, including hand washing, surface cleaning, air disinfection, and attention to indoor ventilation, after vaccination (Table 5). In fact, the HCW's frequencies of hand washing, desk cleaning, and air disinfection increased by an average of 2.5, 0.4, and 8.3%, respectively, after vaccination. The detailed relationship between personal protective behaviors and different population groups (physician, nurse, administrative, cleaner) was shown in Supplementary Table 5. They also paid more attention to indoor ventilation after vaccination. More HCWs wore N95 respirators (24.9% more), face shields (28.1% more), and goggles (21.0% more) after vaccination. No matter what type of HCWs is, vaccination not only eased COVID-19-related fears but also eliminated worry in some HCWs (Supplementary Tables 5, 6). Only 9.5% (127/1,332) of the HCWs expressed that they had no fear of being infected before vaccination, but this percentage increased to 13.5% (180/1,332) after vaccination. Moreover, 21.5% (286/1,332) of the HCWs reported that the pandemic had a large (“more” + “very much”) negative impact on how they treated patients before vaccination, but this percentage decreased to 8.6% (115/1,332) after vaccination.

**Table 5:** Personal protective behaviors of healthcare workers before and after vaccination (*n* = 1,332).

**Personal protective behavior**		**Before**	**After**	***p*** **-value**
		**vaccination**	**vaccination**	
Hand washing times per day		12.2	12.5	0.008
Surface cleaning times per day		2.8	2.9	<0.001
Air disinfection times per day		2.4	2.6	<0.001
Attention to indoor ventilation^a^		3.43	3.60	<0.001
Rates of personal protective measures	Surgical masks	-	-	0.128
	N95 respirators	17.3%	21.6%	<0.001
	Face shields	11.4%	14.6%	<0.001
	Protective clothing	-	-	0.069
	Goggles	8.1%	9.8%	0.015
	Gloves	-	-	0.289
Fear of being infected^b^		2.74	3.04	<0.001
Negative impact on treatment^c^		2.65	2.23	<0.001

a *On a scale of 1 to 5 where 1 indicates very little attention paid to indoor ventilation and 5 indicates a high level of attention paid to indoor ventilation.*

b *On a scale of 1 to 5 where 1 indicates that the respondent was very afraid of being infected with SARS-CoV-2 and 5 indicates that the respondent had no fear of infection during the pandemic.*

c *On a scale of 1 to 5 where 1 indicates that the pandemic had no negative impact on the respondent's treatment of patients and 5 indicates that the pandemic had an extremely negative impact on the respondent's treatment of patients*.

## Discussion

During the ongoing COVID-19 pandemic, HCWs have been the most susceptible group to COVID-19 because of their high rate of contact with both confirmed patients and potentially infected individuals. To prevent the spread of infection, HCWs in many countries adopted the highest level of personal protective measures (e.g., wearing N95 respirators, face shields, gloves, and goggles) (18). When developing efficient COVID-19 control plans, hospitals benefit from knowledge about HCW's personal protective behaviors, their understanding of COVID-19 transmission routes, and changes in their protective behaviors after vaccination.

HCWs have more knowledge about COVID-19 than the general public. Close-contact transmission routes, including both large-droplet and short-range airborne transmission, are considered as the most important routes of SARS-CoV-2 transmission (19, 20). Of these, the short-range inhalation route is the predominant route (21). Although the surface touch (i.e., fomite) and large-droplet routes might also play a role in transmission, their contribution is mostly considered insignificant (22).

Knowledge about COVID-19 is a direct determinant of personal protective behaviors. In our study, 98.0 and 91.8% of 1,499 HCWs believed that large-droplet and short-range airborne transmission, respectively, were possible SARS-CoV-2 transmission routes. Although some studies of indoor outbreaks have shown that long-range airborne transmission is a dominant route, especially in settings with poor ventilation (23, 24), only 20.0% (300/1,499) of the HCWs believed that this was a possible route of SARS-CoV-2 transmission. Accumulating evidence has demonstrated that fomite transmission plays an insignificant role in SARS-CoV-2 transmission (25, 26); however, 66.5% (997/1,499) and 43.0% (645/1,499) of that HCWs believed that SARS-CoV-2 could be transmitted via the short-range and long-range fomite routes, respectively. No gender differences were identified with respect to knowledge about possible transmission routes, consistent with the findings of studies in other countries (27).

Most of the HCWs believed close contact to be a dominant factor in SARS-CoV-2 transmission; consistently with this, 98.7% (1,480/1,499) of the HCWs wore surgical masks or N95 respirators in hospitals, despite the lack of a serious pandemic situation in China at the time of the survey. Our study found that compared with female HCWs, male HCWs had a higher vaccination rate (92.8%, 348/375) but a lower hand washing rate. Studies in other countries have shown that the level of precautionary behavior during COVID-19 pandemic is higher among women than among men (28), but we found no such gender difference among HCWs in hospitals. As of July 12, 2021, the World Health Organization recommends that “vaccinated persons should continue to adhere to public health and social measures and IPC measures. Targeted continuous masking should be implemented in clinical areas of health facilities in areas with known or suspected sporadic transmission.” (29). Similarly, the National Health Commission (30) recommends the use of N95 masks in similar situations.

In addition to the perceived severity of COVID-19, vaccination coverage and efficiency are important considerations driving the stringency of COVID-19 prevention and control measures. After vaccination, the restriction of physical distancing has been found to be relaxed by 36% to 78% (31). As of the end of November, 2021, a total of 7.4 billion vaccine doses have been administered (https://covid19.who.int/). HCWs have been the first-priority group for vaccination in many countries (32). The vaccination coverage of HCWs in England was 89% on February 5, 2021 (33). As of March 2021, the COVID-19 vaccination coverage of healthcare personnel working in long-term care facilities in the United States was highest among physicians (75.1%) and lowest among aides (45.6%) (34). Our survey in Chinese HCWs revealed that 91.6% (417/455) of the physicians and 88.0% (549/624) of the nurses were vaccinated (as of the end of June 2021), and 95.9% (1,438/1,499) of the HCWs believed that the vaccine was effective or highly effective. High confidence among HCWs in vaccination efficacy is important to minimize the negative impact of the pandemic on the HCW's treatment of patients. The average prevalence of COVID-19 vaccination hesitancy in HCWs worldwide has been reported to be 22.5% (35). In comparison, our study showed that only 13.0% (195/1,499) of the HCWs in China's hospitals had no intention to be vaccinated. Furthermore, more female HCWs, individuals with a larger median age, and those with higher number of years of working experiences, intended to accept the COVID-19 (36). However, our results identified no association between the intention to revaccinate in case of vaccination failure and personal attributes of gender and age. Specifically, nurses had a stronger intention to get revaccinated in case of vaccination failure than physicians. In previous studies, 36, 75, 76, and 70% of HCWs in the United States, France, Belgium, and Canada, respectively, reported willingness to take a vaccine as soon as it became available (12, 37). A strong intention to get vaccinated is important for the reduction of infection risks during future waves of COVID-19. In the United States, the vaccination rate was found to be almost exactly inverse to the education level of healthcare staff (38), which was consistent with our findings.

Recently, some SARS-CoV-2 variants (e.g., alpha and delta) have emerged and spread rapidly, and these variants have become the main sources of COVID-19 cases in many countries (e.g., India). If vaccination leads people to relax their protective behaviors, new waves of infection could emerge as the current vaccines may be less effective against such variants (15).

The most important finding of our study is that there is no significant relaxation of personal protective behaviors among Chinese HCWs after vaccination. Our survey was performed on June 30, 2021, just before a new wave of COVID-19 infection in China that could possibly be traced to airport cleaners infected while cleaning a Russian flight cabin on July 10, 2021 (39). Since February 2021, the local transmission of COVID-19 in China had mostly been well controlled (Supplementary Figure 1). However, since July 20, 2021, the infection has spread to more than a dozen provinces, regions, and municipalities (40). China had largely managed to keep imported infections from causing major local outbreaks since the country successfully controlled the domestic spread of SARS-CoV-2 during the first phase of the pandemic. Our previous study showed that university students in China relaxed their personal protective behaviors after vaccination (41). In contrast, this study revealed that the personal protective behaviors of HCWs were sustained after vaccination. Such sustained protective behaviors among HCWs would likely facilitate the reduction of infection risks in hospitals facing the recent new surge of infection from Nanjing to elsewhere in China (40). Although our study revealed sustained personal protective behaviors among Chinese HCWs, it appears that loopholes still exist as a large COVID-19 outbreak occurred in Zhengzhou's Sixth People's Hospital in late July (42).

This study has some limitations. First, the survey could have been affected by response bias because we relied on self-reported data. Second, our data presented may be subject to some confounding factors, but we did not give a comprehensive analysis on confounding factors because of large amount of combinations of parameters. In this study, almost all parameters collected from the questionnaires are independent, and we only analyzed the relationship between each demographic data and personal protective behaviors. Third, personal protective behavior after vaccination may depends on the pandemic situation, which could have led to error. Finally, the number of respondents of cleaner and administrative is limited, which may lower the accuracy of the result for these two types of population.

## Conclusion

Our survey revealed that 89% of the 1,499 Chinese HCWs included in our study had been vaccinated and that 87% of the 1,332vaccinated HCWs expressed that they would get revaccinated in case of vaccination failure. They believed that the large-droplet and short-range airborne routes were the two most likely SARS-CoV-2 transmission routes. Furthermore, the HCWs retained their personal protective behaviors after vaccination. This behavior would facilitate the reduction of infection risks during future waves of COVID-19 due to variants (e.g., delta) in hospitals.

## Data Availability Statement

The original contributions presented in the study are included in the article/Supplementary Materials, further inquiries can be directed to the corresponding author/s.

## Author Contributions

NZ, HL, JX, and YL conceived the study. NZ, LL, TJ, XL, DM, ZB, and LF collected the data. NZ, HL, and BS analyzed the data. NZ prepared the tables and figures. NZ and YL wrote the paper. NZ, HL, PX, JX, and YL made constructive amendments. NZ and HL contributed equally to this work. All authors reviewed the paper.

## Funding

This study was supported by the Natural Science Foundation of China (grant number 52108067) and by a GRF grant (no. 17202719) from the Hong Kong Research Grants Council.

## Conflict of Interest

The authors declare that the research was conducted in the absence of any commercial or financial relationships that could be construed as a potential conflict of interest.

## Publisher's Note

All claims expressed in this article are solely those of the authors and do not necessarily represent those of their affiliated organizations, or those of the publisher, the editors and the reviewers. Any product that may be evaluated in this article, or claim that may be made by its manufacturer, is not guaranteed or endorsed by the publisher.
