# Supplementary material for: COVID-19 Vaccination Did Not Change the Personal Protective Behaviors of Healthcare Workers in China
Source: Front Public Health. 2021 Dec 21;9:777426. doi: 10.3389/fpubh.2021.777426 (PMC8724136; doi:10.3389/fpubh.2021.777426)
Supplement: Supplementary file 1 [file Data_Sheet_1.docx]

**Supplementary Information**

**Table S1**. Demographic data of all respondents

| **Personal information** | **No. of respondents** |
| --- | --- |
| **Gender, n (%)** | n = 1,499 |
| Male | 375 (25.0%) |
| Female | 1124 (75.0%) |
| **Age, n (%)** | n = 1,499 |
| ≤25 years | 225 (15.0%) |
| 26–35 years | 646 (43.1%) |
| 36–45 years | 384 (25.6%) |
| >45 years | 244 (16.3%) |
| **Education level, n (%)** | n = 1,499 |
| Senior middle school or lower | 165 (11.0%) |
| University/junior college | 1088 (72.6%) |
| Master’s or higher | 246 (16.4%) |
| **Annual income (CNY), n (%)** | n = 1,499 |
| ≤50,000 | 455 (30.4%) |
| 50,001–100,000 | 529 (35.3%) |
| 100,001–200,000 | 357 (23.8%) |
| >200,000 | 158 (10.5%) |
| **Occupation, n (%)** | n = 1,499 |
| Physician | 455 (30.4%) |
| Nurse | 624 (41.6%) |
| Administrative agent | 136 (9.1%) |
| Cleaner | 34 (2.3%) |
| **Department, n (%)** | n = 1,499 |
| High-risk department^1^ | 447 (30.3%) |
| Others | 1058 (69.7%) |
| **Hospital classification, n (%)** | n = 1,499 |
| Grade 3A | 354 (23.6%) |
| Grade 3B and 3C | 170 (11.3%) |
| Grade 2 | 707 (47.2%) |
| Grade 1 or below | 268 (17.9%) |
| **Years of work experience, n (%)** | n = 1,497 |
| <5 | 430 (28.7%) |
| 5–10 | 296 (19.8%) |
| 10–20 | 439 (29.3%) |
| >20 | 332 (22.2%) |
| **Vaccination status, n (%)** | n = 1,499 |
| Vaccinated | 1332 (88.9%) |
| Not vaccinated | 167 (11.1%) |
| **Spatial distribution by province** | Figure S1 |

^1^High-risk departments include respiratory, ophthalmology and otorhinolaryngology, anesthesiology, infectious disease, general, and ICU departments.

**Table S2.** Personal protective behaviors of healthcare workers by hospital classification

| **Hospital parameter** | **Hand washing (times/day)** | **Surface disinfection**  **(times/day)** | **Air disinfection**  **(times/day)** | **Ventilation**  **(times/day)** |
| --- | --- | --- | --- | --- |
| **Hospital classification** | p < 0.001 | p = 0.864 | p = 0.053 | p = 0.383 |
| Grade 3A and higher | 11.4 | - | - | - |
| Grade 3B and 3C | 11.6 | - | - | - |
| Grade 2 | 13.8 | - | - | - |
| Grade 1 and lower | 9.4 | - | - | - |

**Table S3.** Percentage distribution of personal protective measures adopted by healthcare workers by demographic characteristics

| **Personal attribute** | **Surgical mask** | **N95 respirator** | **Face shield** | | **Protective clothing** | **Goggles** | | **Gloves** | |  |
| --- | --- | --- | --- | --- | --- | --- | --- | --- | --- | --- |
| **Total** | 92.7% | 17.3% | 11.4% | | 15.0% | 8.1% | | 50.8% | |  |
| **Gender^1^** | p = 0.657 | p = 0.065 | p = 0.871 | | p = 0.132 | p = 0.215 | | p = 0.168 | |  |
| **Age (years)** | p = 0.188 | p = 0.004 | p < 0.001 | | p < 0.001 | p = 0.203 | | p = 0.379 | |  |
| ≤25 | - | 26.1% | 19.3% | | 24.6% | - | | - | |  |
| 26–35 | - | 15.8% | 9.9% | | 14.7% | - | | - | |  |
| 36–45 | - | 15.5% | 12.7% | | 13.0% | - | | - | |  |
| >45 | - | 16.0% | 5.8% | | 10.2% | - | | - | |  |
| **Education** | p < 0.001 | p < 0.001 | p = 0.176 | | p = 0.005 | p = 0.024 | | p = 0.004 | |  |
| High school or lower | 79.9% | 29.5% | - | | 18.1% | 10.7% | | 42.3% | |  |
| University/junior college | 94.6% | 15.9% | - | | 16.1% | 8.7% | | 53.6% | |  |
| Master’s or higher | 93.1% | 15.3% | - | | 7.9% | 3.7% | | 44.0% | |  |
| **Annual income (CNY)** | p = 0.19 | p < 0.001 | p = 0.003 | | p = 0.007 | p = 0.178 | | p = 0.012 | |  |
| ≤50,000 | - | 24.1% | 14.7% | | 20.0% |  | | 45.1% | |  |
| 50,001–100,000 | - | 14.1% | 7.3% | | 13.7% |  | | 54.5% | |  |
| 100,001–200,000 | - | 15.4% | 13.6% | | 13.0% |  | | 54.4% | |  |
| >200,000 | - | 13.8% | 10.9% | | 10.1% |  | | 45.7% | |  |
| **Occupation** | p < 0.001 | p = 0.009 | p = 0.120 | | p = 0.934 | p = 0.590 | | p = 0.174 | |  |
| Physician | 92.6% | 18.2% | - | | - | - | | - | |  |
| Nurse | 97.6% | 12.0% | - | | - | - | | - | |  |
| Administrative staff | 87.7% | 19.7% | - | | - | - | | - | |  |
| Cleaner | 90.3% | 25.8% | - | | - | - | | - | |  |
| **Years of work experience, n = 1,332** | p = 0.674 | p = 0.001 | p = 0.024 | | p = 0.008 | p = 0.105 | | p = 0.101 | |  |
| <5 | - | 22.8% | 13.9% | | 17.8% | - | | - | |  |
| 5–10 | - | 18.8% | 11.4% | | 16.3% | - | | - | |  |
| 10–20 | - | 13.6% | 12.4% | | 15.4% | - | | - | |  |
| >20 | - | 11.8% | 6.1% | | 8.2% | - | | - | |  |
| **Hospital classification** | p = 0.001 | p = 0.197 | | p = 0.937 | p = 0.698 | | p = 0.520 | | p = 0.028 | |
| Grade 3A/3A+ | 92.1% | - | | - | - | | - | | 47.2% | |
| Grade 3B/3C | 90.8% | - | | - | - | | - | | 58.6% | |
| Grade 2 | 95.3% | - | | - | - | | - | | 52.7% | |
| Grade 1 and lower | 87.9% | - | | - | - | | - | | 45.2% | |
| **Department** | p < 0.001 | p < 0.001 | | p < 0.001 | p < 0.001 | | p = 0.001 | | p = 0.041 | |
| High-risk | 87.5% | 27.4% | | 19.0% | 23.8% | | 12.2% | | 55.3% | |
| Others | 94.7% | 13.5% | | 8.5% | 11.6% | | 6.5% | | 49.0% | |

**^1^**Because more than 97% of nurses and more than 70% of cleaners and technicians were female, we only considered physicians and administrative staff when analyzing the correlation between gender and personal protective measures.

**Table S4.** Understanding of the possible transmission routes of COVID-19 among healthcare workers by personal attributes

| **Personal attribute** | **Large droplet** | **Short-range airborne** | **Long-range airborne** | **Short-range fomite** | **Long-range fomite** | **Fecal–oral** | **Blood** | **Mother–baby** | **Mosquito** | **Food and water** |
| --- | --- | --- | --- | --- | --- | --- | --- | --- | --- | --- |
| **Total** | 98.0% | 91.7% | 20.0% | 66.5% | 43. 0% | 50.5% | 29.3% | 22.6% | 19.1% | 31.1% |
| **Gender** | p = 0.137 | p = 0.628 | p = 0.760 | p = 0.014 | p = 0.444 | p = 0.175 | p = 0.677 | p = 0.092 | p = 0.074 | p = 0.022 |
| Male | - | - | - | 61.3% | - | - | - | - | - | 26.4% |
| Female | - | - | - | 68.2% | - | - | - | - | - | 32.7% |
| **Age** | p = 0.032 | p = 0.019 | p < 0.001 | p < 0.001 | p = 0.048 | p = 0.107 | p = 0.009 | p = 0.036 | p = 0.158 | p = 0.188 |
| ≤25 years | 97.3% | 87.1% | 28.9% | 55.1% | 35.1% | - | 36.9% | 28.4% | - | - |
| 26–35 years | 98.9% | 91.6% | 22.4% | 66.6% | 43.2% | - | 30.5% | 23.5% | - | - |
| 36–45 years | 98.2% | 94.3% | 16.1% | 68.0% | 44.8% | - | 25.3% | 20.6% | - | - |
| >45 years | 95.9% | 92.6% | 11.5% | 74.6% | 47.1% | - | 25.4% | 18.0% | - | - |
| **Education** | p < 0.001 | p < 0.001 | p = 0.013 | p = 0.301 | p = 0.018 | p = 0.463 | p = 0.003 | p = 0.001 | p < 0.001 | p = 0.001 |
| High school or lower | 93.3% | 82.4% | 18.8% | - | 35.2% | - | 36.4% | 30.3% | 29.7% | 33.3% |
| University/junior college | 98.4% | 92.7% | 21.7% | - | 42.8% | - | 30.0% | 23.2% | 19.8% | 33.1% |
| Master’s or higher | 99.2% | 93.9% | 13.4% | - | 49.2% | - | 21.5% | 15.0% | 9.3% | 21.1% |
| **Occupation** | p = 0.169 | p = 0.010 | p = 0.435 | p = 0.176 | p = 0.016 | p = 0.345 | p = 0.001 | p < 0.001 | p < 0.001 | p = 0.027 |
| Physician | - | 94.5% | - | - | 50.8% | - | 22.9% | 22.9% | 11.6% | 26.2% |
| Nurse | - | 94.1% | - | - | 41.0% | - | 28.8% | 28.8% | 24.5% | 34.5% |
| Administrative staff | - | 86.8% | - | - | 44.9% | - | 36.0% | 36.0% | 25.7% | 34.6% |
| Cleaner | - | 91.2% | - | - | 41.2% | - | 47.1% | 47.1% | 41.2% | 32.4% |
| **Years of work experience** | p = 0.357 | p = 0.004 | p < 0.001 | p < 0.001 | p = 0.060 | p = 0.266 | p = 0.021 | p = 0.017 | p = 0.369 | p = 0.644 |
| <5 | - | 88.4% | 23.7% | 58.6% | - | - | 33.3% | 24.7% | - | - |
| 5–10 | - | 91.1% | 24.8% | 67.3% | - | - | 31.5% | 26.2% | - | - |
| 10–20 | - | 94.9% | 17.3% | 68.8% | - | - | 25.1% | 20.3% | - | - |
| >20 | - | 94.0% | 10.2% | 74.8% | - | - | 25.2% | 16.9% | - | - |
| **Annual income (CNY)** | p = 0.025 | p = 0.011 | p < 0.001 | p = 0.141 | p = 0.001 | p = 0.434 | p < 0.001 | p < 0.001 | p < 0.001 | p = 0.001 |
| ≤50,000 | 96.5% | 88.4% | 27.0% | - | 42.4% | - | 36.7% | 28.1% | 25.5% | 36.9% |
| 50,001–100,000 | 98.5% | 92.6% | 19.3% | - | 38.0% | - | 30.8% | 24.4% | 24.4% | 31.9% |
| 100,001–200,000 | 98.3% | 94.4% | 15.1% | - | 45.9% | - | 19.6% | 15.1% | 15.1% | 25.8% |
| >200,000 | 100.0% | 93.0% | 13.3% | - | 55.1% | - | 24.7% | 17.7% | 11.4% | 24.1% |
| **Hospital classification** | p = 0.119 | p < 0.001 | p = 0.277 | p = 0.512 | p = 0.094 | p = 0.050 | p = 0.313 | p = 0.403 | p = 0.081 | p = 0.024 |
| Grade 3A and higher | - | 91.0% | - | - | - | 51.1% | - | - | - | 25.1% |
| Grade 3B and 3C | - | 94.1% | - | - | - | 47.1% | - | - | - | 29.4% |
| Grade 2 | - | 94.3% | - | - | - | 53.5% | - | - | - | 34.2% |
| Grade 1 and lower | - | 84.7% | - | - | - | 44.0% | - | - | - | 32.1% |
| **Hospital department** | p = 0.053 | p = 0.095 | p = 0.569 | p = 0.905 | p = 0.326 | p = 0.766 | p = 0.407 | p = 0.419 | p = 0.947 | p = 0.433 |

**Table 5A.** Personal protective behaviors of physicians before and after vaccination (n = 455)

| **Personal protective behavior** | | **Before vaccination** | **After vaccination** | **p value** |
| --- | --- | --- | --- | --- |
| Hand washing times per day | | - | - | 0.764 |
| Surface cleaning times per day | | 2.40 | 2.46 | 0.004 |
| Air disinfection times per day | | 1.95 | 1.95 | 0.049 |
| Attention to indoor ventilation^1^ | | 3.39 | 3.55 | <0.001 |
| Rates of personal protective measures | Surgical masks | - | - | 0.467 |
|  | N95 respirators | 18.23% | 23.02% | 0.003 |
|  | Face shields | 14.87% | 18.71% | 0.003 |
|  | Protective clothing | - | - | 0.056 |
|  | Goggles | - | - | 0.297 |
|  | Gloves | - | - | 0.691 |
| Fear of being infected^2^ | | 2.88 | 3.12 | <0.001 |
| Negative impact on treatment^3^ | | 2.71 | 2.29 | <0.001 |

^1^ On a scale of 1 to 5 where 1 indicates very little attention paid to indoor ventilation and 5 indicates a high level of attention paid to indoor ventilation.

^2^ On a scale of 1 to 5 where 1 indicates that the respondent was very afraid of being infected with SARS-CoV-2 and 5 indicates that the respondent had no fear of infection during the pandemic.

^3^ On a scale of 1 to 5 where 1 indicates that the pandemic had no negative impact on the respondent’s treatment of patients and 5 indicates that the pandemic had an extremely negative impact on the respondent’s treatment of patients

**Table 5B.** Personal protective behaviors of nurses before and after vaccination (n = 624)

| **Personal protective behavior** | | **Before vaccination** | **After vaccination** | **p value** |
| --- | --- | --- | --- | --- |
| Hand washing times per day | | 12.0 | 12.2 | 0.020 |
| Surface cleaning times per day | | 2.94 | 3.01 | <0.001 |
| Air disinfection times per day | | 2.39 | 2.67 | <0.001 |
| Attention to indoor ventilation^1^ | | 3.44 | 3.64 | <0.001 |
| Rates of personal protective measures | Surgical masks | 97.63% | 96.17% | 0.046 |
|  | N95 respirators | 12.02% | 16.39 | 0.001 |
|  | Face shields | - | - | 0.086 |
|  | Protective clothing | - | - | 0.500 |
|  | Goggles | - | - | 0.077 |
|  | Gloves | - | - | 0.606 |
| Fear of being infected^2^ | | 2.65 | 2.99 | <0.001 |
| Negative impact on treatment^3^ | | 2.68 | 2.24 | <0.001 |

**Table 5C.** Personal protective behaviors of administrative before and after vaccination (n = 136)

| **Personal protective behavior** | | **Before vaccination** | **After vaccination** | **p value** |
| --- | --- | --- | --- | --- |
| Hand washing times per day | | - | - | 0.103 |
| Surface cleaning times per day | | 3.07 | 3.18 | <0.001 |
| Air disinfection times per day | | 3.11 | 3.06 | <0.001 |
| Attention to indoor ventilation | | 3.43 | 3.58 | <0.001 |
| Rates of personal protective measures | Surgical masks | - | - | 0.090 |
|  | N95 respirators | 19.67% | 25.41% | <0.001 |
|  | Face shields | - | - | 0.058 |
|  | Protective clothing | - | - | 0.785 |
|  | Goggles | - | - | 0.114 |
|  | Gloves | - | - | 0.257 |
| Fear of being infected | | 2.94 | 3.17 | <0.001 |
| Negative impact on treatment | | 2.49 | 2.13 | <0.001 |

**Table 5D.** Personal protective behaviors of cleaners before and after vaccination (n = 34)

| **Personal protective behavior** | | **Before vaccination** | **After vaccination** | **p value** |
| --- | --- | --- | --- | --- |
| Hand washing times per day | | - | - | 0.065 |
| Surface cleaning times per day | | 2.58 | 2.61 | <0.001 |
| Air disinfection times per day | | 3.29 | 3.48 | <0.001 |
| Attention to indoor ventilation | | 3.68 | 3.84 | <0.001 |
| Rates of personal protective measures | Surgical masks | - | - | 0.225 |
|  | N95 respirators | 6.56% | 4.92% | <0.001 |
|  | Face shields | - | - | 0.064 |
|  | Protective clothing | - | - | 0.789 |
|  | Goggles | - | - | 0.078 |
|  | Gloves | - | - | 0.257 |
| Fear of being infected | | 2.10 | 3.00 | <0.001 |
| Negative impact on treatment | | 2.26 | 2.10 | <0.001 |

**Table S6.** Personal psychology of healthcare workers on COVID-19 prevention before and after vaccination

| **Psychology, n = 1,332** | **Before vaccination** | **After vaccination** | **p value** |
| --- | --- | --- | --- |
| **Fear of being infected, n (%)** | **-** | **-** | <0.001 |
| Very much | 185 (13.9%) | 146 (11%) |  |
| More | 422 (31.7%) | 284 (21.3%) |  |
| Neutral | 403 (30.3%) | 450 (33.8%) |  |
| Less | 196 (14.7%) | 272 (20.4%) |  |
| Very little | 126 (9.5%) | 180 (13.5%) |  |
| **Negative impact on treatment of patients, n (%)** | **-** | **-** | <0.001 |
| Very little | 208 (15.6%) | 307 (23%) |  |
| Less | 405 (30.4%) | 556 (41.7%) |  |
| Neutral | 433 (32.5%) | 355 (26.6%) |  |
| More | 220 (16.5%) | 88 (6.6%) |  |
| Very much | 66 (5%) | 26 (2%) |  |

**Figure S1.** Daily numbers of confirmed COVID-19 cases in mainland China (<https://ourworldindata.org/coronavirus/country/china>).
